# Assessment of Yield and Nutritional Quality of Crop Residues at Different Harvesting Phases and Feeding Priorities in Amhara Region, Ethiopia

**DOI:** 10.1002/vms3.70715

**Published:** 2026-03-09

**Authors:** Malede Birhan Atanaw

**Affiliations:** ^1^ Collage of Veterinary Medicine and Animal Sciences Department of Animal Science University of Gondar Gondar Ethiopia

**Keywords:** crop residue, cutting phases, nutritional quality, yield estimation

## Abstract

This study aimed to estimate the biomass yield and quality of crop residues at different harvesting stages used for animal feed. A longitudinal trend study was conducted to assess changes in crop residue availability across districts over time. A total of 135 feed samples (45 sub‐samples were taken in each district) were collected, from Dembya, Gondar Zuria and Mecha at three harvesting phases. Phase 1 (side‐by‐side cutting with grain harvest), Phase 2 (during threshing, the amount of residue remaining after grain separation) and Phase 3 (from storage or feeding troughs). Samples were collected between December 2023 and February 2024, focusing on residues from teff, sorghum, maize, finger millet and vetch. The result in Dembya, teff straw showed significantly higher (*p* < 0.05) dry matter (93.38%), crude protein (5.93%), neutral detergent fibre (71.99%), in vitro dry matter digestibility (57.84%) and ash content (11.47%). Finger millet quality varied significantly (*p* < 0.05) in DM, CP, NDF, ash and IVDMD, except for ADF, HEM and ADL in certain districts. Dembya had higher CP and IVDMD contents compared to other districts. Teff straw was a primary feed resource, yielding 4.0, 3.8 and 2.96 tons per hectare in Dembya, Gondar Zuria and Mecha, respectively, while vetch straw yields were 3.45, 1.33 and 0.6 tons per hectare. Farmers prioritized feeding plowing oxen and lactating cows during the first and third quarters of the year. In conclusion, harvesting crop residues early improves their nutritional quality, especially when paired with better storage and ensiling methods. In the study area, crop residues are more suitable as supplementary rather than primary feed. Future research should assess animal performance, such as productivity and weight gain, when fed various crop residues.

## Graphical abstract

In Dembya, Gondar Zuria and Mecha districts, cereal residues particularly teff, maize, sorghum, finger millet and vetch straws were identified as the most significantly available livestock feeds (*p* < 0.05). Among these, teff straw from Dembya exhibited superior quality, with 93.4% dry matter (DM), 5.93% crude protein (CP), 11.5% ash and 57.8% in vitro dry matter digestibility (IVDMD). Overall, Dembya recorded the highest nutritional quality, leading in CP (8.7%) and IVDMD (58.5%), followed by Mecha with 7.6% CP and 55.2% IVDMD levels supportive of optimal rumen microbial growth.

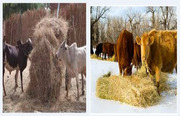

## 1 Introduction

In Ethiopia, livestock farming is a cornerstone of agriculture, providing meat, milk and supplemental income for smallholders (Boloche and Wato 2022). The country's substantial cereal crop production generates significant amounts of crop residues (CRs), which serve as a valuable feed resource for livestock. In fact, CRs are the second most important feed source after grazing lands within Ethiopia's crop‐livestock mixed production system (Adesogan et al. 2025).

However, effective utilization remains underdeveloped due to mishandling and limited awareness on feed improvement strategies (Worku et al. 2023). Globally, CRs account for more than half of dry matter (DM) from harvests, often exceeding the nutrient content of fertilizers used in developing nations (Berhanu et al. 2018).

In Sub‐Saharan Africa, cereal straws serve multiple purposes, including feed (63%), fuel (20%), construction (10%) and bedding (7%) (Jara and Abagisa 2021). In mixed crop‐livestock (MCL) systems, prevalent among smallholder dairy farmers, these residues are vital for livestock feeding and income generation (Berazneva et al. 2018). As arable land expands and grazing areas shrink, reliance on CR increases, limiting the potential for improving animal productivity (Seyoum Bediye, Terefe, Walelegne, Berhane Lakew, et al. 2020).

Since the mid‐1970s, research has focused on enhancing the use of fibrous by‐products from various crops, leading to significant advancements (Alemu et al. 2019). However, many residues, including cereal straws, often have low nutritional value due to poor palatability and digestibility, restricting livestock weight gain when used as untreated roughage (Salles et al. 2019). Without interventions to improve feed quality, these residues provide inadequate intake as the primary roughage feed source (Nyambo et al. 2018).

In the Amhara region, CR production is important for livestock feed, particularly during the dry season. Dominant crops such as teff, maize, sorghum and wheat yield substantial residues, but their nutritional quality is often compromised by improper handling and insufficient treatment methods (Worku et al. 2023). Teff straw (TS) yields range from 2.96 to 4.0 tonnes per hectare, with significant variation among districts like Dembya, Gondar Zuria (GZ) and Mecha (Gebremariam and Belay 2023). Vetch straw (VS) yields are lower, ranging from 0.6 to 3.45 tonnes per hectare, further impacting on feed availability (Degefu and Milkias 2020). The nutritional quality of CR in the Amhara region is concerning; for instance, TS has crude protein (CP) levels as low as 3%–5%, which are insufficient for ruminant supplementations (Alemu et al. 2019).

Addressing quality degradation during harvesting and storage is essential for enhancing livestock productivity. Conservation practices such as proper drying and storage, along with chemical or biological treatments, hold promise for improving residue feed quality and maximizing the use of these resources (Seyoum Bediye, Terefe, Walelegne, Berhane Lakew, et al. 2020).

Despite these insights, important research gaps remain. CRs generally have low protein and energy but high fibre content, reducing their feed value (Hussein et al. 2024). While other researcher explored the bioenergy potential of these residues, regional variations in management and feeding practices have yet to be fully addressed (Tolessa 2023). In addition, current management strategies often lack comprehensive evaluations of residue yield and nutritional quality, particularly when compared to legume residues (Dejene et al. 2024). Therefore, to bridge these gaps, this study evaluates the yield and nutritional quality of CRs at different harvest stages and feeding priorities in the Amhara region is very important to improve livestock productivity and resource use efficiency.

## 2 Research Methodology

### 2.1 Research Site Description

The study was conducted in three agro‐ecological districts in the Amhara region of Ethiopia, including Dembya, Gondar zuria and Mecha, located in the central Amhara highlands. These districts are situated approximately 197 km Northwest, 129 km Northwest and 35 km Southwest of Bahir Dar, the regional capital city, respectively, and the altitude of these areas ranges from 1800 to 2600 Meters above sea level. The region experiences bimodal rainfall, with an average annual precipitation of approximately 800–1200 mm. The main rainy season in all the three districts, extend from June to September, while a smaller wet season starts from April to May. The average temperatures in the highlands range from 10°C to 25°C, with cooler conditions typically found at higher elevations (Alemayehu et al. 2021). Humidity levels are generally moderate, often ranging from 60% to 80%, particularly during the rainy seasons. This climatic context is essential for understanding the agricultural practices and the impacts on crop residue (CR) availability and quality in the region (Alemayehu et al. 2024).

### 2.2 Research Design

A longitudinal trend survey was conducted to assess changes in CR availability over time at different locations in each specific districts. Primary data were collected on selected crop types for yield and quality measurements of the samples on various harvesting phases in three districts. Samples from the same crop types were collected three times from three different locations and pooled into a single sub‐sample for analysis. The residues from each location were combined into composite sub‐samples and analysed for yield and quality evaluation.

A focused group discussion was conducted with farmer representatives from each PA (the smallest administrative unit) across the three districts, selected from key informants. The discussion aimed to gather detailed information on which types of animals were prioritized for CR feeding across different seasons and quarters of the year. It provided insights into how feed management practices varied with seasonal changes and the functional roles of animals throughout the year.

During the dry season, CRs become essential due to limited pasture, with feeding priority given to productive animals such as lactating cows, sick animals and plowing oxen. In the rainy season, the availability of fresh pasture reduces the need for residues, which are often stored for future use during periods of scarcity.

### 2.3 Sampling Techniques and Data Collection Procedure

The study was conducted in three districts Dembya, Gondar zuria and Mecha based on rainfall, animal distribution, dominant crops and feeding practices. A total of 135 pooled CR samples (45 per district) were collected, consisting of nine sub‐samples per crop type from five major CRs in each district. These samples, taken from various farms but within the same crop species, were designed to represent the agricultural and livestock diversity of the region. Quantitative data on CRs were gathered over time to assess bio mass yield and quality at different harvesting phases. Samples were pooled into composite sub‐samples for yield and quality analysis, while seasonal yields, feeding priorities, conservation strategies and feeding practices in each district were also evaluated. Feed samples were collected during and after the feeding of harvested CR at each kebele (the smallest administrative unit) in the study districts.

### 2.4 The Three Phases of Crop residues Sample Collection Procedure

*Phase 1*: Crop residues samples were collected side by side with the food grain harvest (HPR‐1)
*Phase 2*: Crop residues samples were collected during grain threshing (HPR‐2)
*Phase 3*: Crop residues conserved samples were collected the feeding troughs or from the storage (CCR‐3) /HPR‐3)

Farmers' feeding plans for livestock are organized into four quarters: first quarter (Q1) (July–September), second Quarter (Q2) (October–December), third quarter (Q3) (January–March) and fourth quarter (Q4) (April–June), with distinct functions of animals for each period. Feeding strategies prioritized animal needs during different times of the year. The main crops in the research areas are teff straw (TS), sorghum stover (SS), maize stover (MS), finger millet (FM) and vetch (VS), which are commonly used as animal feed.

### 2.5 Quantification of Crop Residues for Yield Measurements

To estimate the DM generated from primary CRs, grain yield data were converted into biomass yield using specific multipliers: 1.5 for wheat, barley and wheat; 1.2 for field pea, faba bean and linseed (Gashaw and Defar 2017). For maize, a multiplier of 2 was applied, while other cereal crops used a multiplier of 1.7 (Wonchesa et al. 2018). It is estimated that approximately 10% of CR production is lost during feeding or repurposed for other uses (Tamrat 2020). However, this estimate does not account for feed obtained from crop leftovers, unavoidable field losses or alternative uses, which could amount to as much as 30% of total agricultural residues (Blümme et al. 2020)

### 2.6 Feed Sample Collection for Chemical Analysis

Feed samples were dried at 105°C, weighed and reweighed to determine DM and moisture content. Samples were analysed for organic matter (OM), CP and ash following (AOAC 2005). Nitrogen (N) was measured, and CP was calculated by multiplying nitrogen contents (N) by 6.25. NDF, ADF and ADL were analysed using (P. V. Van Soest et al. 1991) methods, with ash‐adjusted NDF obtained by burning at 550°C. Hemicellulose was calculated as (NDF − ADF). In vitro dry matter digestibility (IVDMD) was assessed using the modified Tilley and Terry method. Statistical comparisons were conducted with Tukey's and Dunnett's tests, with significance at *p* < 0.05.

For data analysis, the following statistical model was employed.

*Y_i_* = *μ* + *l_i_* + *e_ij_*,
where;
*Y_i_* = quality and quantity of crop residue available in the area
*μ* = Overall mean effect of the quality and the quantity of the residues
*l_i_* = the effect of the *i*th area
*e_ij_* = random error

## 3 Results

In the study area, cereal crop residues, including teff, maize, sorghum, FM and vetch, were the most readily available for animal feed. The availability of each type of agricultural residue varied significantly (*p *< 0.05) across the three districts of Dembya, Gondar zuria and Mecha districts as shown in Table 1.

**TABLE 1:** Feed constituents of teff straw collected in the three study districts.

	Chemical ingredients of the crop residues (%)								
Sites	SCP	DM	CP	NDF	ADF	ADL	HEM	ASH	IVDMD
Dembya (45)	HPR‐1	91.75^c^	6.57^a^	67.43^c^	43.36^c^	12.02^cd^	24.92^b^	12.59^a^	62.22^a^
	HPR‐2	93.25^b^	5.12^c^	71.70^b^	46.78^b^	14.13^b^	25.0^b^	10.71^b^	57.13^b^
	CCR‐3	95.13^a^	4.11^d^	74.85^a^	48.68^a^	15.72^a^	26.17^a^	9.64^c^	54.18^c^
	Mean	93.38^bd^	5.93^cb^	71.99^bd^	47.94^be^	13.96^e^	24.05^bc^	11.47^d^	57.84^bd^
	Std.	0.24	0.606	0.40	0.61	0.413	0.641	0.037	0.035
	Sig.	^*^	^*^	^*^	NS	NS	NS	^*^	^*^
Gondar Zuria (45)	HPR‐1	88.41^c^	7.14^a^	65.32^c^	42.36^c^	10.75^b^	22.96^b^	12.41^a^	64.13^a^
	HPR‐2	92.34^b^	5.31^b^	68.26^b^	45.18^b^	14.26^b^	23.06^b^	10.81^b^	55.71^b^
	CCR‐3	94.13^a^	3.31^c^	71.23^a^	47.88^a^	15.32^a^	23.35^a^	8.98^c^	51.25^c^
	Mean	91.63^bd^	5.25^bd^	68.27^db^	45.20^bd^	14.42^bc^	22.79^bc^	10.73^bd^	56.66^bd^
	Std.	0.041	0.031	0.05	0.026	0.33	0.14	0.006	0.008
	Sig.	^*^	^*^	^*^	NS	NS	NS	^*^	^*^
Mecha (45)	HPR‐1	89.18^c^	7.23^a^	69.36^a^	41.48^c^	11.18^b^	27.88^a^	13.59^a^	61.25^a^
	HPR‐2	92.13^b^	5.95^b^	64.92^b^	45.29^b^	14.41^b^	19.63^b^	10.67^b^	55.63^b^
	CCR‐3	95.27^a^	4.43^c^	60.66^c^	49.39^a^	15.19^a^	11.27^c^	7.56^c^	43.21^c^
	Mean	92.19^be^	5.87^bd^	64.98^be^	45.31^be^	13.95^bc^	19.93^d^	10.61^bd^	53.36^de^
	Std.	0.043	0.044	0.03	0.32	0.071	0.7	0.038	0.011
	Sig.	^*^	^*^	^*^	NS	NS	NS	^*^	^*^

*Note*: Means with different superscript letters within a column and across the row show significant level (*p* ≤ 0.05).

Abbreviations: NS = not significant; SCP = Sample collection process; HPR = harvesting phases of the residues; CCR = conserved crop residue. DMB = dry matter bases; CP = crude protein; NDF = neutral detergent fibre; ADF = acid detergent fibre; ADL = acid detergent lignin; HEM = hemicellulose; IVDMD = in vitro dry matter digestibility; DMB = dry matter bases of the crop residues.

* *p* ≤ 0.05).

**p ≤ 0.01).

The research findings demonstrated that teff straw (TS) from the Dembya district exhibited significantly higher (*p* < 0.05) levels of key nutritional components, including DM, CP, neutral detergent fibre (NDF), ash and IVDMD. The mean values were recorded as 93.38% for DM, 5.93% for CP, 71.99% for NDF, 11.47% for ash and 57.8% for IVDMD. Similar result showed on TS samples from Mecha and GZ districts displayed comparable results. In Mecha, the values for DM, CP and NDF were depicted as 92.2%, 5.87% and 64.98%, respectively, while in Gondar zuria (GZ), the corresponding values were 91.6% for DM, 5.25% for CP and 68.27% for NDF (Table 1).

Among the three districts, Dembya and Mecha were found to have higher CP content (5.93% and 5.87% as compared to GZ. This suggests that the nutritional quality of TS varies geographically, with Dembya and Mecha districts offering better CP content, which is a critical factor for the nutritional value of livestock feed (Table 1).

Significantly higher (*p* < 0.05) CP content of sorghum stover (SS) in Dembya district suggests better nutritional quality compared to other study areas Table 2. This difference may be attributed to variations in soil fertility, climate or cultivation practices, which influence nutrient uptake in the residue. The elevated NDF concentration in sorghum residue across all the three harvest dates in Dembya, recorded at 71.4%, reflects a higher structural carbohydrate content, likely indicating more fibrous plant material. This could be due to environmental conditions promoting more lignification or delayed harvesting, which often increases fibre levels as plants mature. Thus, the SS in Dembya and Gondar zuria (GZ) districts exhibited higher CP (7.1% and 6.24%) and NDF (71.4% and 70.2%) contents, indicating better nutritional quality. In contrast, the lowest CP content was recorded in Mecha district at 4.1%, making it the least favourable among the studied areas in terms of nutritional value Table 2.

**TABLE 2:** Chemical compositions of sorghum stover in the three districts of the study area.

		Chemical ingredients of the crop residues (%)							
Sites	SCP	DM	CP	NDF	ADF	ADL	HEM	ASH	IVDMD
Dembya	HPR‐1	89.0^a^	10.33^a^	66.74^a^	42.28^a^	11.06^a^	24.46^a^	13.59^a^	65.19^a^
	HPR‐2	93.58^b^	6.41^b^	70.97^b^	47.29^b^	13.24^ab^	23.68^ab^	11.68^b^	58.34^b^
	CCR‐3	94.68^c^	4.43^c^	76.61^c^	52.11^c^	14.27^ac^	24.50^ac^	10.60^b^	47.28^c^
	Mean	93.42^bd^	7.06^d^	71.44^bd^	47.19^bd^	12.85^abc^	24.22^abc^	11.77^bd^	56.93^ed^
	Std.	0.031	0.001	0.003	0.041	0.61	0.72	0.03	0.003
	Sig.	^*^	^*^	^*^	^*^	NS	NS	NS	^*^
G/Zuria	HPR‐1	89.11^a^	7.89^a^	67.82^a^	41.98^a^	18.98^a^	25.84^a^	11.34^a^	55.84^a^
	HPR‐2	93.10^b^	6.22^c^	59.92^b^	44.18^b^	21.73^b^	15.74^b^	9.85^b^	44.98^b^
	CCR‐3	94.25^c^	4.54^b^	61.62^c^	47.35^c^	25.96^c^	14.27^c^	7.82^c^	39.26^c^
	Mean	92.15^bd^	6.24^cd^	63.12^d^	44.50^bd^	22.22^bd^	14.63^cd^	9.67^bd^	46.69^d^
	Std.	0.037	0.012	0.026	0.044	0.022	0.048	0.039	0.007
	Sig.	^*^	^*^	^*^	^*^	NS	NS	^*^	^*^
Mecha	HPR‐1	90.14^a^	8.76^a^	60.15^a^	46.69^a^	20.11^a^	13.46^a^	13.12^a^	67.32^a^
	HPR‐2	93.33^b^	5.26^b^	66.32^b^	49.72^b^	22.21^ab^	15.6^b^	11.36^b^	61.25^b^
	CCR‐3	95.17^bc^	4.12^c^	70.15^c^	53.54^c^	24.31^ac^	16.61^c^	8.34^c^	55.13^c^
	Mean	92.88^bd^	6.05^bd^	65.54^bd^	49.98^bd^	22.21^ad^	23.02^bd^	10.94^bd^	61.23^bd^
	Std.	0.029	0.002	0.037	0.041	0.174	0.034	0.039	0.038
	Sig.	^*^	^*^	^*^	NS	NS	NS	^*^	^*^

*Note*: Mean with different superscript letters within a column are different at (*p* < 0.05).

Abbreviations: NS = not significant; SCP = Sample collection process; HPR‐1‐3 = harvesting phase of the residues from 1 to3; DM = dry matter; CP = crude protein; NDF = neutral detergent fibre; ADF = acid detergent fibre; ADL = acid detergent lignin; HEM = hemicellulose; IVDMD = in vitro dry matter digestibility; DMB = dry matter bases of the crop residues.

* *p* < 0.05.

**p < 0.01.

In‐vitro dry matter digestibility (IVDMD) of SS in the study area demonstrated a highly significant variance (*p* < 0.05) across all the three districts. Dembya and Mecha districts had the highest IVDMD values, at 56.9% and 61.2%, respectively, indicating superior digestibility and better quality for animal feed. These results suggest that SS from Dembya and Mecha is more suitable for livestock due to its higher digestibility compared to other study areas (Table 2).

In the Dembya district, the CP content of FM straw was found to be significantly higher (*p* < 0.05), with a mean value of 6.4%, contributing to its high nutritional value (Table 3). In addition, in Mecha area, the mineral and NDF contents of FM residues were also highly significant (*p* < 0.05), with average values of 12.5% for ash and 67.9% for NDF, respectively, as presented in Table 3., which emphasize the superior feed potential of FM residues in these areas. While numerical differences in the DM content of FM straw were observed across the three harvesting stages, these differences were not statistically significant (*p* > 0.05) across the three districts.

**TABLE 3:** The feed ingredient of finger millet used for animal feeding in the districts.

		Chemical ingredients of the crop residues (%)							
Study sites	SCP	DM	CP	NDF	ADF	ADL	HEM	ASH	IVDMD
Dembya	HPR‐1	91.14	7.628^a^	62.42^a^	41.24^a^	13.44^a^	21.18^a^	12.75^a^	54.84^a^
	HPR‐2	93.42	6.34^b^	64.78^b^	44.75^b^	14.98^b^	20.03^a^	11.01^b^	49.93^b^
	CCR‐3	94.89	5.15^c^	66.24^c^	49.15^c^	15.77^c^	17.08^b^	10.11^b^	42.62^c^
	Mean	93.15	6.37^bd^	64.48^ad^	45.05^bd^	14.73^bd^	19.43^ad^	11.29^bc^	49.13^bd^
	Std.	0.11	0.06	0.03	0.15	0.32	0.42	0.02	0.06
	Sig.	NS	^*^	^*^	NS	NS	NS	NS	^*^
G/Zuria	HPR‐1	88.36	6.26^a^	50.86^a^	26.64^a^	13.21^a^	24.22^a^	12.44^a^	48.86^a^
	HPR‐2	91.65	4.64^b^	53.14^b^	32.73^b^	15.38^b^	20.40^b^	10.58^b^	45.15^b^
	CCR‐3	94.37	3.11^c^	61.75^c^	39.71^c^	18.82^c^	22.04^a^	9.13^c^	39.65^c^
	Mean	91.46	4.67^bd^	55.25^d^	33.03^bd^	15.80^bd^	22.22^a^	10.72^bd^	44.55^bd^
	Std.	0.41	0.033	0.003	0.006	0.044	0.026	0.011	0.014
	Sig,	NS	^*^	^*^	^*^	NS	NS	^*^	^*^
Mecha	HPR‐1	91.66	5.573^a^	63.76^a^	42.12^a^	12.52^a^	21.64^b^	14.06^a^	55.84^a^
	HPR‐2	93.74	4.78^b^	66.36^b^	41.26^a^	14.26^b^	25.10^a^	12.71^b^	50.15^b^
	CCR‐3	95.13	4.11^b^	72.52^c^	45.63^b^	15.62^c^	26.90^a^	10.631^c^	46.62^c^
	Mean	93.51	4.82^b^	67.88^bd^	43.01^ac^	14.13^bd^	24.55^bc^	12.47	50.87^bd^
	Std.	0.31	0.04	0.002	0.1	0.36	0.2	0.12	0.03
	Sig.	NS	^*^	^*^	NS	NS	NS	^*^	^*^

*Note*: Mean with different superscript letters within a column are different at (*p* < 0.05).

Abbreviations: NS = not significant; SCP = Sample collection process; HPR‐1–3 = harvesting phase of the residues from 1 to 3; DM = dry matter; CP = crude protein; NDF = neutral detergent fibre; ADF = acid detergent fibre; ADL = acid detergent lignin; HEM = hemicellulose; IVDMD = in vitro dry matter digestibility; DMB = dry matter bases of the crop residues.

* *p* < 0.05.

^**^ *p* < 0.01.

This lack of significant difference could be attributed to the fact that FM is harvested after the stacks and grains are fully dried both the stalk and the food grain. As a result, the dry matter content of the straw remains consistent across the three harvesting stages found in Table 3.

Based on research findings from the Dembya district, the contents of CP, acid detergent fibre (ADF), minerals and IVDMD in vetch straw (VS) exhibited significant differences (*p* < 0.05), with the mean values of 16.3%, 46.55%, 14.6% and 66.9%, respectively indicated in Table 4

**TABLE 4:** The feed constituents of vetches collected on the three districts the study area.

		Chemical ingredients of the crop residues (%)							
Sites	SCP	DM	CP	NDF	ADF	ADL	HEM	ASH	IVDMD
Dembya	HPR‐1	89.19^c^	18.37^a^	56.74^b^	42.25^c^	13.11^c^	14.49^c^	16.58^a^	71.32^a^
	HPR‐2	92.88^b^	16.37^b^	60.97^c^	47.28^b^	14.09^b^	13.69^b^	14.67^b^	66.17^b^
	CCR‐3	94.16^a^	14.29^c^	65.61^a^	50.13^a^	16.13^a^	15.48^a^	12.53^c^	63.14^c^
	Mean	92.08^bd^	16.34^bd^	61.11^d^	46.55^bd^	14.44^bd^	14.56^ad^	14.59^bd^	66.88^bd^
	Std.	0.011	0.007	0.033	0.025	0.041	0.029	0.001	0.022
	Sig.	^*^	^*^	^*^	^*^	NS	NS	^*^	^*^
G/Zuria	HPR‐1	90.23^c^	14.79^a^	55.81^c^	38.93^c^	11.96^a^	16.88^c^	11.32^a^	61.41^a^
	HPR‐2	93.62^b^	11.21^b^	61.71^b^	42.04^b^	13.71^a^	19.67^b^	9.53^b^	55,80^b^
	CCR‐3	95.11^a^	9.85^d^	66.13^a^	45.55^a^	15.11^a^	20.58^a^	7.83^c^	51.96^c^
	Mean	92.98^db^	11.95^bd^	61.22^bd^	42.17^b^	13.93^a^	19.04^b^	9.56^d^	45.79^d^
	Std.	0.024	0.027	0.003	0.031	0.12	0.018	0.026	0.002
	Sig.	^*^	^*^	^*^	NS	NS	NS	^*^	^*^
Mecha	HPR‐1	91.36^c^	16.92^a^	62.28^c^	40.99^c^	10.93^c^	21.29^b^	15.22^a^	61.41^a^
	HPR‐2	94.26^b^	13.22^b^	67.13^b^	44.32^b^	13.64^b^	22.81^b^	13.22^b^	54.91^b^
	CCR‐3	95.78^a^	11.81^c^	71.37^a^	47.67^a^	16.27^a^	23.30^ab^	10.85^c^	48.87^c^
	Mean	93.8^bd^	13.98^bd^	66.93^bd^	44.09^bd^	13.61^bd^	22.83^b^	13.10^bd^	55.06^bd^
	Std.	0.032	0.041	0.042	0.005	0.011	0.063	0.044	0.003
	Sig.	^*^	^*^	^*^	NS	NS	^*^	^*^	^*^

In addition, the NDF and hemicellulose contents of VS in the Mecha district also showed statistically significant variation (*p* < 0.05), with measured values of 66.9% and 22.8%, respectively, as depicted in Table 4 below.

The research findings indicate that the Dembya district recorded the highest average values for CP, IVDMD and ash content, followed by the Mecha district (Table 4). This variation may be attributed to Dembya's mid‐latitude location, which receives ample moisture and experiences optimal ambient temperatures. In addition, the soil fertility in both study areas likely contributes to these higher nutrient values by promoting better plant growth and development.

The current result showed that there are no significant differences (*p* > 0.05) in the mean values of CP contents of MS showed in three districts (Dembya, GZ and Mecha), with the average values of 7.58%, 7.91% and 7.2%, respectively (Table 5). However, there is a significant decline (*p* < 0.05) in CP and IVDMD content as MS residue was harvested at later stages (HPR‐1 to HPR‐3). Explicitly, in Dembya district, CP contents was decreased from 9.9% at the first stage to 5.6% at the third stage, and IVDMD decreased from 68.9% at the first stage to 57.2% at the last stages of harvest correspondingly (Table 5).

**TABLE 5:** The chemical composition of maize stover used for animal feed in Dembya district.

		Chemical ingredients of the crop residues (%)							
Sites	SCP	DM	CP	NDF	ADF	ADL	HEM	ASH	IVDMD
Dembya	HPR‐1	88.35^a^	9.89^a^	66.7^a^	47.28^a^	12.06^a^	19.42^a^	13.588^a^	68.93^a^
	HPR‐2	91.58^b^	7.29^b^	71.0^b^	50.11^b^	13.24^a^	20.89^b^	11.671^b^	59.65^b^
	CCR‐3	94.67^c^	5.56^c^	76.6^c^	57.21^c^	14.27^a^	19.39^c^	10.056^b^	57.18^c ^
	Mean	91.87^bd^	7.58^bd^	71.4^bd^	51.53^bd^	13.19^a^	22.57^ad^	11.77^b^	61.53^d^
	Std.	0.27	0.17	0.13	0.17	0.77	0.46	0.042	0.015
	Sig.	^*^	^*^	^*^	NS	NS	^*^	^*^	^*^
G/Zuria	HPR‐1	88.37^a^	10.3^a^	65.9^a^	47.71^a^	11.18^a^	18.19^a^	12.22^a^	65.23^a^
	HPR‐2	90.38^b^	8.18^b^	71.4^b^	45.11^b^	14.85^b^	26.29^b^	9.51^b^	59.18^b^
	CCR‐3	93.11^c^	5.22^c^	74.2^c^	46.29^c^	17.32^c^	27.91^b^	7.72^c^	51.74^c^
	Mean	90.62^bd^	7.91^d^	70.5^d^	43.7^d^	14.45^bd^	19.78^b^	9.8^bd^	58.72^d^
	Std.	0.035	0.81	0.01	0.026	0.42	0.91	0.008	0.033
	Sig.	^*^	^*^	^*^	^*^	NS	NS	^*^	^*^
Mecha	HPR‐1	90.18^a^	11.0^a^	63.4^a^	43.9^a^	13.44^a^	19.50^a^	10.59^a^	61.18^a^
	HPR‐2	94.21^b^	6.44^b^	66.4^b^	49.5^b^	14.48^b^	16.90^b^	8.67^b^	55.25^b^
	CCR‐3	96.78^c^	4.13^c^	73.7^c^	46.84^c^	14.73^b^	26.87^c^	8.85^b^	49.82^c^
	Mean	94.06^bd^	7.2^d^	67.8^b^	46.71^c^	14.22^b^	24.44^a^	8.7^b^	55.42^b^
	Std.	0.042	0.01	0.02	0.011	0.027	0.041	0.026	0.001
	Sig.	^*^	^*^	^*^	^*^	NS	NS	NS	^*^

Similarly, in Gondar zuria (GZ) and Mecha districts, the CP content decreased as the harvesting stages progressed from the first to the third stage across all residue types and districts. In contrast, the NDF content increased significantly from Phase 1 to 3 for all crop types, indicating greater lignification and a decline in forage quality with delayed harvest. These findings suggest that postponing harvest has negatively affects forage quality, as reflected by the reduced CP and IVDMD (Table 5).

**FIGURE 1:**
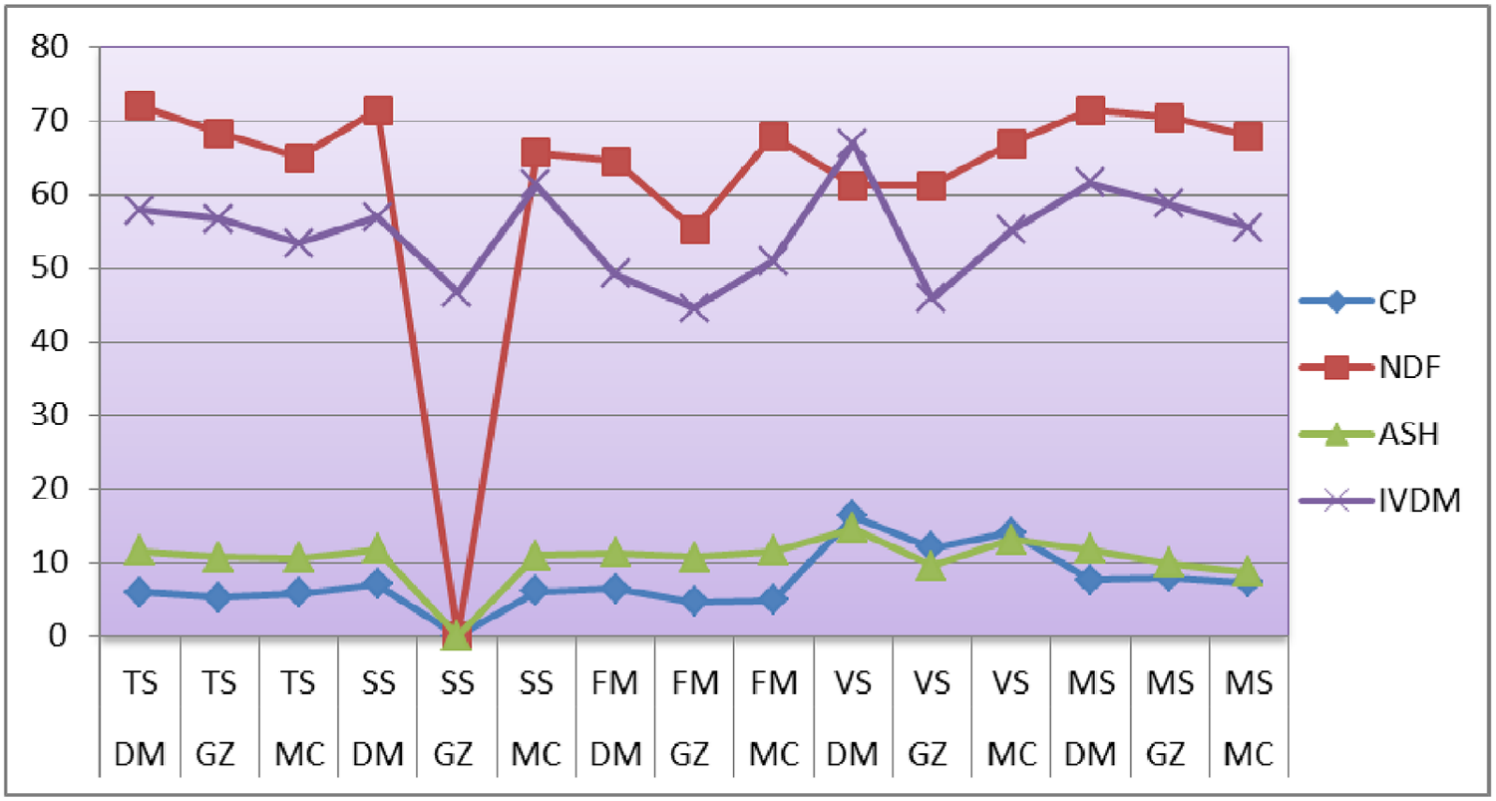
The average values of forage quality ingredient estimated in the study area. Key: TS = teff straw, SS = sorghum straw, FM = finger millet, VS = vetch straw, MS = maize stover, DM = Dembya, GZ = Gondar Zuria, MC = Mecha, CP = crude protein, NDF = neutral detergent fibre, IVDMD = in vitro dry matter digestibility.

Figure 1 clearly depicts the average values of CP, NDF, ash content and IVDMD across five CRs in the three study districts. During the quantitative data collection phase, a strong priority was given by farmers in each district regarding animal feeding based on their specific functions and health status.

The results indicates in Dembya, Gondar (GZ) and Mecha districts, farmers prioritize feeding of the CRs to plowing oxen and lactating cows during the first and third quarters of the year (Table 6). In the first quarter, 82.7% of farmers in Dembya, 88.4% in GZ and 91% in Mecha prioritized feeding for plowing oxen at the first quarter of the year. Similarly, during this period, 71.9% of farmers in Dembya, 84.1% in GZ and 69.9% in Mecha prioritized feeding CRs to lactating cows again in the first quarter of the year. These findings underscore the importance of CRs for maintaining the health of working oxen and the production performance of lactating dairy cows, particularly during critical agricultural seasons when forage becomes scarce (Table 6).

**TABLE 6:** Feeding ranks on observational study feeding priorities in each quarter of the years.

Animal types	Study area	Feeding in the ranking order	Proportional feeding	Season of the year			
				1Q	2Q	3Q	4Q
Plowing oxen	Dembya	1st rank	0.827	Yes	—	—	—
		2nd rank	0.15	—	Yes	—	—
		3rd rank	0.023	—	—	Yes	—
	G/Zuria	1st rank	0.884	Yes	—	—	—
		2nd rank	0.086	—	Yes	—	—
		3rd rank	0.034	—	—	Yes	—
	Mecha	1st rank	0.91	Yes	—	—	—
		2nd rank	0.056	—	Yes	—	—
		3rd rank	0.034	—	—	Yes	—
Sick animal	Dembya	1st rank	0.51	—	Yes	—	—
		2nd rank	0.24	Yes	—	—	—
		3rd rank	0.22	—	—	Yes	—
		4th rank	0.03	—	—	—	Yes
	G/Zuria	1st rank	0.61	—	Yes	—	—
		2nd rank	0.31	Yes	—	—	—
		3rd rank	0.06	—	—	Yes	—
		4th rank	0.02	—	—	—	Yes
	Mecha	1st rank	0.56	—	Yes	—	—
		2nd rank	0.28	Yes	—	—	—
		3rd rank	0.14	—	—	—	Yes
		4th rank	0.02	—	Yes	—	—
L/cows	Dembya	1st rank	0.69	Yes	—	—	—
		2nd rank	0.18	—	Yes	—	—
		3rd rank	0.07	—	—	Yes	—
		4th rank	0.06	—	—	—	—
	G/Zuria	1st rank	0.61	—	Yes	—	—
		2nd rank	0.31	Yes	—	—	—
		3rd rank	0.06	—	—	Yes	—
		4th rank	0.02	—	—	—	Yes
	Mecha	1st rank	0.70	Yes	—	—	—
		2nd rank	0.19	—	Yes	—	—
		3rd rank	0.09	—	—	Yes	—
		4th rank	0.02	—	—	—	Yes

*Note*: 1Q, 2Q, 3Q and Q = first, second, third, fourth quarter (January, February and March), (April, May and June), (July, August and Sept) and (October, November and December) respectively, of the feeding year, Q = Quarter *N* = number of observation, Yes = the farmers are feeding the animal as a priorities at the quarter of the year.

Furthermore, weak and sick animals were prioritized feeding the CRs in each respective quarter of the year. In the second quarter, 51.4%, 61% and 55.5% of the available residues in Dembya, G/Zuria and Mecha districts, respectively, were allocated to feed to sick animals. In general, during the fourth quarter of the year, farmers were not concerned about feeding priority to their animals, as ample green forages were available due to the rainy season and sufficient moisture available in the region. As a result, feeding priorities to the different types of animals were focused on the dry periods of the year.

Farmers prioritize feeding based on the function of the animals like milking and sick animals are given priority to sustain milk production and ensure the health of the animal, while work animals receive more attention during the agricultural season to maintain their strength for fieldwork. The discussion also uncovered variations in feeding practices across different PAs, influenced by access to feed resources, economic priorities and cultural preferences. These findings suggest that policy and extension efforts should consider seasonal‐specific interventions, such as promoting residue storage and feed supplementation during the dry season.

In the three study districts, feeding livestock with various types of CRs near homesteads in the morning and evening were a common practice, particularly during the rainy season.

The study highlights a significant variation in dry matter yield (DMY) produced at different CRs among the three study districts, with a notable contribution from TS in the Dembya district. The production of TS in Dembya district accounted for 59% of the animal feed source derived from this residue, making it the highest proportional contributor in the area (Table 7). The DMY of TS was significantly (*p* < 0.05) higher (4 t/ha) compared to the other districts. GZ and Mecha Districts the dry matter yield produced from TS were considerably lower, with 3.8 and 2.96 t/ha with the proportional contribution of 46% and 31.1% of the feed sources for animal feeding respectively, highlighting a variation in the availability of this feed source across districts.

**TABLE 7:** Forage biomass yield and its relative Contribution of the residues in the study area.

	Dembya (*N* = 45)		Gondar Zuria (*N* = 45)		Mecha (*N* = 45)	
Residues	Mean ± Std. (tonne/ha)	PC	Mean ± Std. (tonne)/ha	PC	Mean ± Std (tonne)/ha	PC
Teff straw	4 ± 0.01^a^	0.59	3.8 ± 0.17^a^	0.46	2.96 ± 0.02^a^	0.31
Sorghum stover	1.6 ± 0.03^b^	0.17	2.0 ± 0.04^b^	0.13	0.64 ± 0.34^c^	0.11
Corn stover	0.46 ± 0.02^d^	—	0.7 ± 0.03^b^	0.12	2.0 ± 0.17^b^	0.44
Finge rmillet	0.5 ± 0.04^b^	0.021	0.8 ± 0.62^b^	0.11	2.22 ± 0.03^b^	0.01
Vetch straw	3.45 ± 0.007^c^	0.157	1.33 ± 0.51^d^	0.11	0.6 ± 0.014^c^	0.02

*Note*: Letters, a, b and c indicates the mean with different superscript letters within a row showed significant different (*p* ≤ 0.05). Abbreviations: PC = proportional contribution, *N* = number respondents; Sdt. = standard deviation, OC = Over all contribution.

In all the study districts, VS had the second‐highest biomass yield produced across the districts, yielding as 3.45 t/ha in Dembya, 1.33 t/ha in GZ and 0.6 t/ha in Mecha districts respectively. Although vetch is a legume crop which has relatively high in CP content as compared to other cereal CRs, its proportional contribution to animal feed resources was relatively low, accounting for 15.7%, 11% and 2.0% in Dembya, GZ and Mecha districts, respectively. This variation in CR yield and proportional contribution to animal emphasizes the differing agricultural practices and resource availability across the three districts, of TS being the dominant feed source in Dembya (Table 7).

### 3.1 Discussion

In Dembya district, SS was found to have a CP concentration of 7.1%, which is still insufficient for supporting optimal rumen fermentation despite being slightly above the typical CP levels of other CRs (Chen et al. 2024). Better soil fertility and moisture in the Dembya district, combined with enhanced competition among crops for nutrient uptake in the soil, and that might contribute SS had a relatively higher CP content compared to other residues (Shinde et al. 2022). Similarly, MS in Dembya recorded a CP content of 7.58%, whereas VS exhibited a significantly higher CP value of 16.34%. This aligns with previous findings, which report that leguminous forages generally have superior CP content compared to grass species (Shi et al. 2020)

The CP content of VS was notably higher across all study regions, with 16.34%, 11.95% and 13.94% in Dembya, GZ and Mecha districts, respectively (Table 8). This higher CP content is attributed to vetch being a leguminous crop, known for its rich protein content, which exceeds the threshold needed for rumen microbial activity and supports better animal productivity (Kumar et al. 2023). In contrast, the majority of the straw residues from other cereals, such as teff, maize and sorghum, had CP levels below 70 g/kg DM, which is far below the necessary protein levels for the function of rumen microbes (P. Van Soest 1982).

**TABLE 8:** Correlation coefficient of chemical composition of crop residue ingredients.

	CP	DM	NDF	ADF	ADL	ASH	HEM	CELL	IVDMD
CP	1.000	−0.156	−0.151	−0.135	0.014	0.166	0.021	−0.116	0.0025
DM		1.000	0.063	0.028	−0.281^*^	−0.082	0.031	0.004	0.012
NDF			1.000	0.775^**^	0.531^**^	−0.052	0.182	0.670^**^	0.314^**^
ADF				1.000	0.603^**^	0.047	−0.303^*^	0.820^**^	−0.531
ADL					1.000	0.089	−0.149	0.510^**^	−0.831^*^
ASH						1.000	−0.201	0.113	−0.082
HEM							1.000	−0.261^*^	−0.231^*^
CELL								1.000	−0.362^*^
IVDMD									1.000

The study also highlighted that most CRs exhibited high NDF levels; exceeding 650 g/kg DM. High NDF levels are typically associated with low digestibility, which can hinder the performance of dairy cows by limiting feed intake and nutrient absorption (Seyoum Bediye, Terefe, Walelegne, Fekadu, et al. 2020). This is consistent with previous studies that found higher NDF content in cereal CRs compared to legume residues like VS (Koura et al. 2016). The higher NDF levels in agricultural residues may be due to the longer maturation period of crops, leading to increased fibre formation and lignification, which reduces their nutritional value for ruminants.

However, VS again stood out, having lower NDF levels and thus being more digestible and better suited for livestock feed. The relatively lower fibre content of VS supports better rumen function and animal performance, compared to the other high‐fibre residues (Seyoum Bediye, Terefe, Walelegne, Berhane Lakew, et al. 2020).

Despite the high NDF content in most CRs, the study noted that the mean IVDMD levels were relatively high, averaging 55% across all three research districts. This suggests that while the residues may not provide sufficient CP, their DM digestibility remains moderately high. However, even with this digestibility, the lack of CP indicates that these residues alone are not sufficient for ruminants, and protein supplementation remains necessary (Shi et al. 2020).

The research highlights that the most common CRs used as livestock feed in the study areas were TS, MS, SS, FM straw and VS. These residues, while abundant, generally provide insufficient CP content for optimal rumen fermentation in ruminants, which typically requires above 7.5% CP for effective microbial activity in the rumen (P. V. Van Soest et al. 1991). In all three research regions, the majority of the CRs had CP levels below this critical threshold, indicating a deficiency in nutritional quality for ruminants.

The findings of the study highlight a significant nutritional challenge for livestock in the three districts, where most CRs, except for VS, fall short in CP content, making them insufficient to support effective rumen fermentation in ruminants. Livestock rely on suffcient CP in their diet to maintain proper rumen function, particularly for the microbes responsible for fibre digestion (Wang 2023). However, the majority of CRs, especially those from the second and third harvests, fall below this threshold. This deficiency results in suboptimal feed that compromises animal productivity.

In conclusion, supplementing low‐quality CRs with protein‐rich feeds such as leguminous forages or commercial protein sources and improve the CRs by a various mechanisms are essential for improving animal health and productivity in the region.

Policy efforts and extension advocacy should promote early harvesting stages, as they offer better forage quality and yield potential in the region as well as at national level.

Further research should prioritize urea treatment, ensiling and other CR improvement techniques, including ammonization, to enhance nutritional value. In addition, on‐farm feeding trials assessing the impact of these strategies on milk production, weight gain and overall livestock performance are critical for supporting sustainable and improve livestock production.

## Author Contributions

The research is done by the author.

## Funding

The author has nothing to report.

## Ethical Statement

The author has nothing to report.

## Conflicts of Interest

The author declares no conflicts of interest.

## Data Availability

The data that support the findings of this study are available on request from the corresponding author. The data are not publicly available due to privacy or ethical restrictions.
